# Translating Research Findings Into Practice: Example of Treatment Services for Adolescents in Managed Care

**Published:** 2006

**Authors:** Stacy Sterling, Constance Weisner

**Affiliations:** Stacy Sterling, M.P.H., M.S.W., is a senior research project manager, and Constance Weisner, Dr.P.H., M.S.W., is an investigator, both in the Division of Research, Kaiser Permanente Northern California, Oakland, California. Dr. Weisner also is a professor in the Department of Psychiatry, University of California, San Francisco, California

**Keywords:** health services research, health care delivery, AOD (alcohol and other drug) use, AODD (AOD disorder) care provider, adolescent drinking, dual diagnosis, alcoholism treatment services research, treatment program, research in practice, research-practice integration (RPI) model, information transfer from research to practice, technology transfer, evidence-based practices/intervention

## Abstract

An important question in the alcoholism treatment field is how research findings can be translated into real-world clinical practice. Researchers have developed a new research–practice integration (RPI) model that can both drive the formulation of studies and new research questions and promote improvements in treatment quality. The hallmark of this model is a collaborative relationship between the key stakeholders in both alcohol and other drug (AOD) treatment and research, including health plan administrators and clinicians, treatment program administrators, psychiatry and primary care departments, patients and their families, purchasers, and researchers. The issue of technology transfer is especially relevant in the realm of adolescent AOD treatment. The implementation and feasibility of the RPI model are illustrated by a case study of a managed health care plan’s treatment services for adolescents with AOD dependence. In this setting, key research findings are being used to shape the plan’s adolescent health services.

Translating research findings into clinical practice is a central aspect of current medical science and a primary focus of health services research. A commonly used term in this context is “research to practice,” which refers to the process of developing appropriate research questions, disseminating the results of such research, and applying those findings to clinical practice. Over the past decade, investigators and clinicians have put much effort into fostering an environment conducive to vigorous technology transfer. In spite of these efforts, however, there is continuing concern in the alcohol and other drug (AOD) treatment fields that much of the growing knowledge of effective treatment is not finding its way into clinical practice. This article provides an overview of an innovative, bidirectional “research–practice integration” (RPI) process and describes an RPI model relevant to the alcohol field. The need for RPI particularly in programs that treat adolescents with alcohol use disorders is addressed, and a case study illustrates how the RPI model is applied to alcoholism treatment for adolescents in a managed care organization.

## Research–Practice Integration

The literature on health care technology transfer suggests that a complex set of factors can shape the relationship between research and clinical practice. Numerous stakeholders are involved with AOD treatment, such as health plan administrators, addiction medicine policymakers, AOD program administrators and clinicians, primary care and psychiatric health care providers, consumers (i.e., patients), and health plan purchasers (e.g., employers). Factors influencing RPI that are related to these various stakeholders include financing issues, organizational culture and attitudes, and lack of infrastructure or expertise. Other factors impacting RPI that may act as barriers to the adoption of new practices based on research findings are more specific to the AOD field. These factors include the stigma associated with AOD use disorders, diverse schools of thought within the treatment community regarding the most appropriate treatment approaches, clinical attitudes, and the unique role of the consumer in the clinical relationship (Backer et al. 1995).

To address these diverse factors, eliminate potential barriers, and thereby enhance RPI, many policymakers and funding organizations have made the dissemination of research findings and the adoption and utilization of proven treatment technologies a high priority. The National Institute on Alcohol Abuse and Alcoholism (NIAAA), the National Institute on Drug Abuse (NIDA), and the Center for Substance Abuse Treatment (CSAT) within the Substance Abuse and Mental Health Administration (SAMHSA), as well as private foundations such as the Robert Wood Johnson Foundation, have each sponsored efforts to accelerate this process. These efforts include the Research to Practice Forums, the Researcher in Residence program, the Connecting Services and Research (CSR) system, the Addiction Technology Transfer Centers (ATTCs), and various conferences and community symposia (U.S. Department of Health and Human Services 2000).

Researchers increasingly are examining deficits in the traditional approach to dissemination of research findings, which involves publishing articles in the scientific literature and making presentations at conferences and symposia (Brown 2000). Other investigators have suggested conceptual models (Simpson 2002) and strategies for facilitating the transfer of new treatment technologies (ATTC Network 2004; Brown 2003), which have resulted in significant insights:

- Backer (1995) determined that organizational readiness for change is a crucial prerequisite for successful technology transfer.
- Sorensen and Clark (1995) described a successful technology transfer strategy that explicitly integrated dissemination activities into a research program.
- The ATTC National Office developed a practical, step-by-step guide to technology transfer activities called *The Change Book* (ATTC Network 2004; Brown 2003).

Despite such efforts to increase the adoption of new treatments, however, research findings related to AOD treatment still are clearly underutilized. With few exceptions, no clinical guidelines have been developed in addiction medicine and AOD treatment, or have achieved the same level of acceptance as similar guidelines in other medical specialty areas. Moreover, interventions that have been shown to be effective in treating AOD use disorders and other addictions have not been widely adopted.

## A Novel RPI Model

Researchers from Kaiser Permanente Northern California and the University of California San Francisco recently developed a collaborative RPI model that is based on published findings, organizational theory, and an understanding of how health plans work. This model draws both on experience in the AOD field (Backer et al. 1986; Brown 2003; Petry 2000; Sorensen and Clark 1995) and on the larger technology transfer literature, incorporating concepts such as social marketing, readiness for change, planned versus responsive and reactive health promotion, and theories of technology diffusion (Backer 1995; Lehman et al. 2002; Lomas 1993; Sobell 1996). The hallmark of this model is a bidirectional relationship between the key stakeholders in both AOD treatment and research (e.g., patients, clinicians, managers, policymakers, and researchers) (see the figure). Depending on the expertise and resources needed, different stakeholder groups take the lead at different stages in the research and RPI process. Thus, the model assumes continuous interaction between the various stakeholder groups, and feedback from them, both in developing research questions and in adopting findings to clinical practice. The ultimate goals are to improve clinical practice, including better patient outcomes, and to generate research questions for further study.

This model is circular and the various stages can be repeated several times. To illustrate the process, one can begin with the development of research questions. Clinically relevant research questions can be derived from many different sources, such as:

- Findings from earlier studies (e.g., on the proven efficacy of new treatments).
- Clinical concerns (e.g., the presence of co-occurring disorders in AOD patients or the rapid rise in the abuse of certain pain medications).
- Changing cultural attitudes (e.g., lessening of sanctions for marijuana use).
- Policy agendas (e.g., efforts by health plans to comply with legislation to ensure that AOD treatment receives the same insurance coverage as treatment for other medical conditions [i.e., parity]).

To develop research questions that are relevant to clinical practice, researchers must work closely with AOD clinicians and program managers and with policymakers in the larger organization. New clinical concerns can emerge at any point—for example, when new evidence-based technologies are developed and must be adapted to real-world settings, or when new client characteristics are identified that raise important clinical questions. Researchers can use these concerns to formulate testable research questions that can lead to generalizable results and to develop appropriate treatment interventions for study, drawing also upon the literature in other related fields, earlier findings, and scientific expertise.

Once research questions have been generated and/or an intervention developed that takes the concerns of the various stakeholder groups into consideration, investigators can design and conduct a study of the effectiveness of this intervention. If the approach studied proves effective (or an epidemiological study provides new information on client populations that helps determine treatment needs), appropriate changes in treatment programs should be implemented. As with the initial development of the intervention, the input of key stakeholders in shaping program services is critical. During the implementation process it is important to maintain the integrity of the original intervention studied while ensuring its feasibility and acceptability in real-world treatment programs.

To ensure that treatment programs will adopt and continue to implement new services and interventions, research staff must work closely with the programs to provide training and technical assistance until the program management and staff feel comfortable and proficient with the new approach. Subsequently, the groups should continue to meet regularly to discuss the ongoing progress of the new services and ensure the successful adoption of the new technology. Finally, feedback from the various stakeholders regarding the overall impact of a particular intervention or policy should be continuously incorporated into the development of new research questions, beginning the cycle of RPI anew.

## Factors Influencing the RPI Model

Central steps in RPI activities in any health care organization are to:

- Identify key decisionmakers and other stakeholders (e.g., health plan administrators, policymakers, AOD program administrators, clinicians, mental health and primary care providers, patients, and health plan purchasers).
- Delineate the interests and concerns of these stakeholders.
- Create potential strategies for addressing these interests and concerns.

As new research questions are generated and RPI activities evolve, the types of stakeholders involved and their relative influence on the process may shift. Moreover, these stakeholder groups will differ across various types of institutions, such as juvenile justice, welfare agencies, and schools. The following discussion focuses on the influence of stakeholder perspectives in the context of a health plan; different sets of factors may become relevant in other settings.

**Figure:**
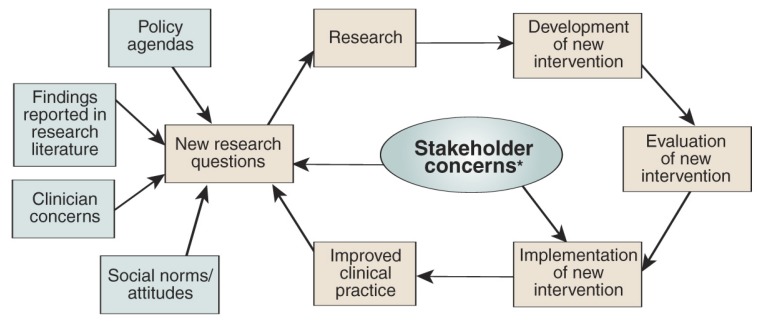
A model of research–practice integration. * Stakeholders include: consumers, program administrators, researchers, primary care and psychiatry providers, AOD clinicians, purchasers/employers, health plan regional/national leadership, and accreditation bodies (e.g., National Committee for Quality Assurance).

### Organizational Factors

RPI efforts to change AOD treatment practices in health care organizations can face significant challenges related to the organization’s administrative infrastructure. These challenges may include competing disease priorities, financial concerns, infrastructure problems, and data collection that is not standardized across programs. For example, health care organizations may spend fewer organizational resources on AOD problems than on disorders such as cardiovascular disease or diabetes. Furthermore, fierce competition in the health care marketplace can result in financial obstacles to the adoption of new technologies, particularly in relation to disorders that receive less attention than others, such as AOD disorders. In addition, health care organizations often lack the management information systems infrastructure necessary to collect and manage research-quality data, and most U.S. AOD programs do not standardize their data collection.

To address these challenges, the RPI model presented here is centered on collaboration among researchers, clinicians, and organizational decisionmakers (e.g., best practices groups or groups of medical department chiefs). According to this model, researchers can provide data on problem prevalence, services utilization, and cost impacts. Clinicians then can use this information to increase organizational decisionmakers’ awareness of the significance and burden of AOD problems, encourage the organization’s support of treatment approaches that are informed by research, and make a “business case” on how providing high-quality, evidenced-based AOD treatment is valuable to the organization. Researchers also can provide technical assistance to the organization to facilitate standardization of data collection and management instruments and generate capacities to gather high-quality patient- and program-level data for future research and program evaluation.

### Program Factors

Concerns about resources—such as staff time, space, and information technology capacity—may impede the adoption of new interventions and technologies in individual treatment programs. Mistrust of or unfamiliarity with the research process, as well as lack of enthusiastic, visible leadership support for studies, also can hinder the RPI process. Accordingly, researchers must work closely with program administrators to promote structural changes that allow new treatment approaches to be studied and implemented. For example, many of the current evidence-based interventions rely on one-to-one interactions between therapist and patient rather than the group-based therapies predominantly used in AOD treatment programs. Therefore, adopting one-on-one approaches may be more complex than simply beginning to offer the intervention and may require major restructuring of programs and possibly an enhancement of staff skills in general. Some programs also may have contracts with purchasers or behavioral health firms to provide a particular standard or model of care, preventing the introduction of new approaches. Finally, some new interventions (e.g., harm reduction models or medications) can pose challenges to long-held beliefs regarding appropriate treatment approaches.

Research teams must maintain ongoing contact with program leadership to encourage active support of research activities, thereby facilitating a “research-friendly” environment in the program. Only in such an environment is it possible to optimize adoption of new successful interventions, study adaptations of evidence-based approaches, and develop new “next-step” research questions. Researchers also must interact with program managers to thoroughly assess the financial impact of any new treatment approach and to identify and target resource barriers.

### Clinical Factors

Clinicians involved in AOD treatment represent perhaps the most crucial stakeholder group in the RPI model presented here. Consequently, most RPI activities traditionally have taken place at the clinical level in the form of treatment manuals and training. At the same time, some of the obstacles to knowledge transfer and utilization are directly related to the role of clinicians. These obstacles include time constraints, ideological differences on the best treatment approaches, lack of familiarity with the research process, failure of traditional modes of information dissemination, and differences in the data needs of clinicians. To help address these obstacles, researchers must be especially careful to involve AOD clinicians in the research process, rather than just presenting them with a new intervention. By addressing long-held concerns about certain treatment approaches (e.g., use of medications) and developing research study designs that take into account the context within which new interventions will take place, researchers enhance not only the feasibility of the study but also the likelihood that the findings will later be adopted.

In the RPI model presented here, researchers work with clinical interest groups, reporting findings and soliciting clinician expertise on the interpretation of study results and the development of further relevant research questions. The technology transfer literature suggests that traditional means of dissemination—through academic journals and conferences—may be ineffective with clinicians (Lamb et al. 1998). Accordingly, this RPI model proposes that research teams instead use a variety of strategies and media to share findings from research studies with clinicians and thus involve them in the research enterprise. These strategies could include interactive small-group seminars and training sessions, teleconferencing, and user-friendly clinical tools, such as screening instruments and client educational materials that use key, understandable statistics.

### Patient Factors

The AOD treatment community has lagged behind other areas of medicine with respect to understanding the role of patients in influencing treatment approaches. Historically, AOD patients and their families rarely have been involved in advocating the development of new therapies or in shaping the treatments available to them. The RPI model described here facilitates patients’ understanding of available treatments and research opportunities, thereby helping to develop the “consumer” status that patients now enjoy in other areas of health care—including the ability to effectively seek out and demand evidence-based treatments (Institute of Medicine 2001, 2005).

From the patients’ perspective, a critical barrier to the transfer of research findings into clinical practice is the overwhelming stigma associated with AOD use disorders. Negative attitudes toward AOD patients from society at large, from treatment providers, and from patients themselves can make it almost impossible for patients to assume the role of health care consumers empowered to demand the highest quality evidence-based treatment (Link et al. 1999). Effective dissemination of research findings and assistance with the interpretation of these findings offer the best answers to this challenge. Accordingly, in this RPI model the research teams provide patients with information from their earlier studies and findings from the broader literature related to AOD treatment effectiveness using media such as newsletters, Web sites, and discussion groups. Focus groups and interviews also can effectively solicit patient concerns and inform future research based on the patients’ experiences with AOD problems and existing treatment.

### Purchasing-Decision Factors

Purchasers of health care services include employers, unions, and other organizations that decide which treatments should be available to their members. Large purchasers frequently are concerned about costs and have misperceptions about the effectiveness of certain treatment approaches, which have been shaped by marketing campaigns. Because insurers are responsive to purchaser demands, this stakeholder group has the power to significantly impact the types of benefits health plans provide. For example, purchasers may demand that health plans limit AOD treatment benefits, and/or they may contract out AOD services to other providers. Conversely, purchasers may insist upon more intensive and costly forms of treatment, such as inpatient treatment, believing that it will prove more effective for their employees or members. These requests may not be related to what is evidence based. AOD program administrators can use the research findings that have been provided to them to educate organizational leaders (and through them, purchasers) about effective treatment interventions and to promote evidence-based treatment interventions.

### Factors Related to Other Health Care Providers

#### Primary Care Providers

Across most health care organizations, primary care providers and AOD treatment providers generally continue to operate independently, with little integration of their services, although growing evidence suggests that such integration can contribute to better outcomes (Weisner et al. 2001; Fleming et al. 1997). This lack of integration can lead to missed opportunities to intervene with patients who could benefit from AOD treatment (Bethell et al. 2001; Singer et al. 1987).

For a variety of reasons—including lack of knowledge or discomfort about AOD use disorders, inadequate clinical tools, time constraints, ignorance of treatment resources, and issues of professional jurisdiction—many primary care providers rarely screen for or discuss AOD use with their patients (American Academy of Pediatrics 2002). As a result, patients at risk for AOD-related problems may not be identified until they exhibit severe symptoms. Researchers can address such barriers by working with primary care providers to identify obstacles to screening and treatment, to develop user-friendly instruments that physicians consider realistic and feasible for use in busy medical settings, and to study the organizational/structural barriers to screening. According to the RPI model presented here, researchers should actively solicit the input of primary care providers on a variety of research activities ranging from research questions to dissemination strategies. Such discussions can enhance the relationships between primary care providers and AOD treatment clinicians on the one hand and primary care providers and researchers on the other, as well as provide a forum in which physicians can suggest questions for further study.

#### Psychiatric Care Providers

Many patients receiving AOD treatment have mental health problems; conversely, many patients with mental health problems have AOD problems (Escobedo et al. 1998; Grella et al. 2001; Hser et al. 2001; Morrison et al. 1993; Rao et al. 2000). In fact, the concept of “dual disorders” has become nearly ubiquitous in both the AOD and psychiatry fields. Although the AOD and psychiatry fields have closer disciplinary ties to each other than to the primary care field, they have exhibited less than optimal collaboration in the past—a state of affairs that often continues to this day (SAMHSA 2002; Institute of Medicine 2005).

Mental health clinicians’ beliefs, training, and behaviors regarding AOD use can constitute important barriers to the effective treatment of patients with AOD-related problems. Historically, mental health providers have not routinely assessed patients for AOD problems (and by the same token, AOD treatment providers have not systematically screened their patients for mental health problems). In addition, ideological differences between the mental health and alcohol fields sometimes have created barriers to effective assessment, referral, and treatment of mental health patients with AOD problems.

To facilitate new interventions, researchers must actively seek to involve mental health clinicians and administrators in the RPI process for AOD treatment. Clinical mental health opinion leaders can drive the process of knowledge utilization. As with primary care providers, researchers should solicit the input of these opinion leaders on the development of research questions relevant to their clinical interests, on strategies for successfully implementing evidence-based practices featuring psychiatric components, and on studies of the effectiveness of integrated treatment approaches. Researchers also should facilitate the information exchange between AOD researchers and clinicians on the one hand and psychiatric clinicians on the other hand by organizing opportunities for multidisciplinary communication and education.

## Research–Practice Integration in AOD Treatment of Adolescents

The RPI model can be particularly important in the area of AOD treatment of adolescents, because RPI has been less well studied in this area, and fewer proven, evidence-based interventions are available for this population. In this case, the RPI model builds on the adolescent treatment literature but also relies on clinicians, patients, and parents to help guide the development of research questions and interventions to be tested.

An increasing number of studies have demonstrated that treatment can be effective for adolescents with AOD-related problems (Booth and Kwiatkowski 1999; Catalano et al. 1990–91; Deas and Thomas 2001; Jainchill 2000). Treatment in a variety of settings (e.g., inpatient, residential, and outpatient) and using various modalities (e.g., 12-step, family systems, and cognitive-behavioral therapy) has been found to reduce both AOD use and problems in other domains (e.g., mental health, educational, and legal problems) (Grella et al. 2001; Hser et al. 2001; Waldron and Kaminer 2004).

Most adolescents who seek AOD treatment also have problems in other areas of their lives. For example, many teenagers in AOD treatment also suffer from mental health problems and/or have experienced family conflicts as well as educational and legal problems. Similarly, experimentation with AODs is relatively common among adolescents, and most of them do not develop AOD abuse or dependence. Accordingly, the assessment, diagnosis, and treatment of AOD-related problems can be more difficult in adolescents than in adults. Furthermore, because adolescent AOD use so often is entangled with other problems, a flexible, integrated approach to treatment is especially appropriate with this population. For these reasons, researchers have tested the application of the RPI model to AOD treatment of adolescents. The following section presents the findings of one case study.

### Case Study of the Application of the RPI Model in AOD Treatment of Adolescents

A study by Sterling and colleagues (2004) applied the RPI model to AOD treatment services for adolescents in a managed health care plan. These researchers examined pathways to treatment, the demographic and epidemiological characteristics of the adolescents entering treatment, and the factors associated with treatment retention and outcome. The treatment programs studied were representative of common AOD treatment approaches for adolescents in the United States, which typically are abstinence based, intensive, structured, and provided on an outpatient basis (Jainchill 2000). (For more information on the sample, program sites, and methodology of the study, see Sterling et al. 2004.) The study involved the participation of all stakeholders outlined in the RPI model.

This study was initiated by the health plan’s adolescent AOD treatment clinicians, who expressed concerns that patients delayed entering treatment until their problems (including mental health problems) had become severe. The clinicians also were interested in examining which patients had better outcomes and which treatment factors were related to improvement. To ensure a positive experience for the participating programs and to optimize the relevance of study findings, the researchers worked with clinicians and program administrators to identify relevant research questions, develop study instruments also appropriate for clinical practice, and design study protocols that would minimally disrupt clinic operations. AOD treatment providers and administrators (as well as adolescent patients and their parents) also participated in extensive qualitative interviews and focus groups that provided background information for understanding the study findings and informed subsequent followup data collection.

As epidemiological data and outcome results from the study have become available, researchers have regularly presented the results to program staff and solicited their opinions on the implications of these findings for treatment services and coordination.

Key findings that have emerged from this study to date include the following:

- Compared with matched control subjects, adolescents entering AOD treatment had significantly higher rates of several psychiatric and medical conditions (Sterling et al. 2004). They also had more legal, educational, and familial problems.
- Adolescents entering treatment reported high levels of AOD consumption as well as AOD abuse and dependence symptoms, which could confirm clinicians’ suggestions that these patients were delaying treatment entry until their problems became severe (Sterling et al. 2004).
- Significant variability existed in the pathways through which these patients entered treatment (Sterling et al. 2004). For example, boys were more likely than girls to have been referred to treatment from the legal system. Only a relatively small proportion of the sample had been referred by one of their health plan’s medical or psychiatric providers.
- Girls were significantly more likely than boys to have received previous mental health treatment (Sterling et al. 2004).
- Integrated services—such as concomitant AOD and psychiatric treatment and delivery of various other services in a single location, also referred to as “one-stop shopping”—improved AOD outcomes (Sterling and Weisner 2005).

Taken together, these findings suggest that adolescents entering AOD treatment have serious problems across several different life domains and that health plan departments likely to deal with these adolescents (i.e., AOD treatment, primary care, and psychiatry departments) could more effectively identify and refer “at risk” patients for treatment. Moreover, structural and clinical changes that increase integration between the involved departments, particularly between the AOD programs and the psychiatry department, could improve outcomes significantly .

The results obtained by Sterling and colleagues (2004) have been used in turn to alter the health plan’s practices for AOD treatment of adolescents in various ways:

- The researchers participating in the study worked closely with the health plan’s program administrators and regional leadership groups (e.g., best practices committees and departmental chiefs groups) to develop programs and policies that can improve interdepartmental integration. For example, findings from this and related studies helped the health plan’s Dual Diagnosis Best Practices Committee to implement a “liaison” initiative, which places a clinician specially trained in dual diagnoses in each AOD treatment and psychiatry clinic to act as a resource for professional staff in these clinics. Similarly, findings from this and other studies have been used at liaison training sessions on co-occurring disorders and integrated treatment.
- The Dual Diagnosis Best Practices Committee also used the study’s prevalence data on comorbidity when discussing resource allocation with the health plan’s leadership, emphasizing the value of providing high-quality services for patients with co-occurring conditions.
- The researchers also are continuing to work with other health plan leadership groups (e.g., pediatric chiefs, pediatric psychiatry chiefs, adolescent AOD treatment coordinating chiefs) to influence adolescent health care policy and practices in the organization (e.g., developing a study that will survey primary care providers to identify barriers to screening and referral of adolescents with AOD problems).

Over the course of this interaction between the researchers and the various stakeholder groups, the stakeholders have become effective clinical partners, suggesting additional research questions that are relevant to their clinical work and the larger field.

## Conclusions

Approaches to integrating research and practice have changed in the past few years in two important ways. First, most traditional RPI models have focused on a unidirectional process of information dissemination—that is, from research to the clinical setting—on the assumption that research questions were developed by the researchers and the results then flowed down to practice. The case example presented here, however, demonstrates the synergy that can develop when clinicians and other stakeholders are brought into the process of developing research questions that can either be new or serve as “next steps” in a clinic’s development of evidence-based interventions.

Second, the traditional RPI process has not involved multiple stakeholders and has focused primarily on conventional forms of technology transfer (i.e., publishing articles and manuals and providing training). In contrast, this model illustrates the importance of considering the wide range of stakeholders involved. Thus, the recent experience of health services researchers has highlighted the importance of a two-way RPI model that, in addition to the research perspective, incorporates knowledge of organizations, financing, and program characteristics, as well as clinician, patient, and environmental factors, using a variety of technology transfer approaches.

The case study presented here was based on the organizational context of a health plan; other considerations would come into play in other organizational settings, such as criminal justice, school, or child welfare settings. In a school setting, for example, successful implementation of an intervention based on enhanced screening and referral of adolescents with AOD problems would involve the funding entity of the school (e.g., community or private organization), the school district, principals, teachers, parents, and students. Even specialized alcoholism treatment programs outside of health plans have analogous stakeholders in their fiscal components and/or larger umbrella organizations that provide funding or play other important roles for some of these programs.

The next stages of health services research on RPI and adolescent AOD treatment should study how stakeholders from different types of agencies (e.g., schools, juvenile justice, welfare) can be involved. This process should identify key strategies that are both relevant across different settings and unique to particular settings. Furthermore, it is important to develop ways to communicate research questions raised by patients, clinicians, and program administrators to researchers. Many researchers in academic settings do not have the benefit of contact with treatment institutions. Therefore, it will be essential to devise ways to link research groups with treatment programs that have similar interests.

The development and implementation of RPI models is an exciting and timely area of health services research, particularly in the AOD treatment field. These models can help identify research questions that assist in developing new interventions or constellations of services, adapt them to real-world clinical practice, and measure their effectiveness.
